# Reduction of Hepatic Fat Content by Dulaglutide for the Treatment of Diabetes Mellitus: A Two‐Centre Open, Single‐Arm Trial

**DOI:** 10.1002/edm2.70021

**Published:** 2024-12-24

**Authors:** Chuanfeng Liu, Yu Xin, Yajing Huang, Lili Xu, Ruizhi Zhou, Yangang Wang, Wei Wang

**Affiliations:** ^1^ Department of Hematology Affiliated Hospital of Qingdao University Qingdao China; ^2^ Department of Endocrinology and Metabolic Diseases Jiaozuo People's Hospital Jiaozuo China; ^3^ Department of Endocrinology and Metabolic Diseases Affiliated Hospital of Qingdao University Qingdao China; ^4^ Department of Radiology Affiliated Hospital of Qingdao University Qingdao China

**Keywords:** GLP‐1, NAFLD, T2DM

## Abstract

**Background:**

With the elevated level of NAFLD prevalence, the incidence of diabetes, hypertension, metabolic syndrome and other diseases is also significantly elevated. GLP‐1RA can exert weight loss, glucose‐lowering effects and various nonglycaemic effects. However, the relationship between quantitative reduction in hepatic fat content and improvement of pancreatic islet function by GLP‐1RA is unclear.

**Methods:**

This trial was a single‐arm open cohort study. A total of 38 patients with T2DM and NAFLD were enrolled in the GLP‐1RA treatment group. The included patients were tested for biochemical and blood glucose levels, adiponectin and FGF21 levels, and liver fat content was measured using MRI. Measure the above indicators again after at least 3 months of GLP‐1RA treatment. Divided into Q1 (average decrease of 0.37%) and Q2 (average decrease of 8.6%) groups based on the degree of reduction in liver fat content.

**Results:**

Q2 group showed an average reduction in liver fat content of 8.6%, a decrease in glycated haemoglobin of 18.17%, a weight loss of 7.29% and an increase in fasting c‐peptide release by 1.03%, 1‐h and 2‐h postprandial c‐peptide release by 28.86% and 18.28% respectively. In contrast, Q1 group had an average reduction in liver fat content of 0.37%, a decrease in glycated haemoglobin of only 6.53%, a weight loss of 3.41%, a decrease in fasting c‐peptide release by 1.91% and an increase in 1‐h and 2‐h postprandial c‐peptide release by 19.18% and 11.66% respectively.

**Conclusion:**

Reduction in liver fat content effectively improves pancreatic islet function secretion, particularly postprandial c‐peptide secretion, especially in the first hour after a meal. This improvement leads to a decrease in glycated haemoglobin levels and promotes better compliance with blood glucose control.

## Graphical abstract

We quantitatively measured the changes in liver fat content before and after GLP‐1RA treatment using liver fat quantification technology. To evaluate the relationship between liver fat content and remission of diabetes.

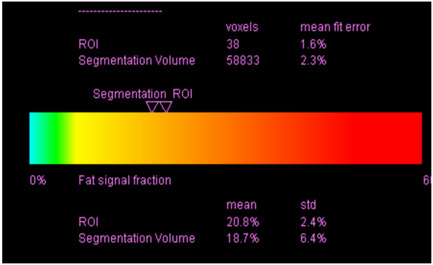

## 1 Introduction

Over the past 50 years, the worldwide prevalence of obesity has increased to epidemic proportions [1]. The obesity epidemic has worsened the burden of metabolic as well as nonalcoholic fatty liver disease (NAFLD). Excessive obesity usually develops slowly over time due to a long‐term positive energy balance. The fatty liver leads to an increased output of triglycerides (TGs) in the hepatic circulation, which in turn leads to ectopic deposition of extrahepatic fat [2]. A meta‐analysis of 86 epidemiological studies indicates that the global prevalence of NAFLD is approximately 25%. This condition is strongly associated with metabolic diseases such as obesity, Type 2 diabetes mellitus (T2DM), hyperlipidaemia, hypertension and metabolic syndrome [3]. NAFLD occurs in about 25% of the population and is closely linked to various metabolic diseases, including obesity, T2DM, hyperlipidaemia, hypertension and metabolic syndrome. As NAFLD progresses, it results in hepatic fat accumulation, hepatocyte ballooning and infiltration of inflammatory cytokines. This progression can lead to nonalcoholic steatohepatitis (NASH), which is associated with an increased risk of all‐cause mortality [4], as well as hepatocellular carcinoma [5] and extrahepatic cancers [6].

Glucagon‐like peptide‐1 (GLP‐1) is primarily secreted by intestinal L cells [7]. It promotes insulin secretion in a glucose‐dependent manner and has widespread expression of GLP‐1 receptors throughout the body, leading to various nonglycaemic effects [8]. These effects include cardiovascular protection [9], blood pressure reduction [10], regulation of lipid metabolism [5], control of gastrointestinal motility and delay in gastric emptying. GLP‐1, when peripherally secreted, does not cross the blood–brain barrier [11]. Only the small amount of GLP‐1 expressed in the nucleus ambiguous acts on the central GLP‐1 receptor. This may be one reason why GLP‐1 affects cognitive function, mood and appetite suppression [11]. GLP‐1 analogues are also approved as first‐line drugs for the treatment of Type 2 diabetes and obesity [12].

Although weight loss is currently recognised as a crucial aspect of alleviating or even reversing diabetes, the quantitative relationship between liver fat reduction and the degree of diabetes improvement remains unclear. Therefore, this study aimed to investigate the relationship between the decrease in liver fat content and the rate of achieving glycaemic control, the recovery of pancreatic function and changes in lipocalin and fibroblast growth factor 21 (FGF21) levels. We examined the degree of liver fat reduction in patients before and after GLP‐1 treatment to explore these relationships.

## 2 Research Objectives and Methods

### 2.1 Design Overview

The trial was a two‐centre, open, single‐arm, 3‐month study comparing the differences in liver fat content and blood glucose levels before and after Dulaglutide 1.5 mg once a week in individuals with T2DM and NAFLD. Recruitment of patients from Rizhao People's Hospital and Qingdao University Affiliated Hospital. Written informed consent was obtained from all participants.

#### 2.1.1 Inclusion and exclusion criteria

Patients with T2DM combined with NAFLD, BMI > 24 kg/m^2^ and 18–80 years of age were initially screened from outpatient clinics or wards to form the treatment group. The diagnosis of NAFLD is achieved through MRI liver fat quantification.

Patients with Type 1 diabetes, acute complications of diabetes, alcoholic fatty liver disease, acute liver and kidney injury, tumours and pregnant women were excluded. Patients need to receive GLP‐1RA medication, therefore patients who receive GLP‐1RA prior to inclusion in the trial will be excluded.

### 2.2 Procedures

After the screening period, general patient data were collected with primary observation indexes including the blood glucose levels and liver fatty content measured by MRI. Secondary observation indexes encompassed pancreatic islet function markers such as fasting c‐peptide, 1‐h c‐peptide and 2‐h c‐peptide, as well as blood glucose levels, blood lipids, urinary protein and liver and kidney function parameters. Additionally, lipocalin and FGF21 were explored as additional indicators.

After 3 months of Dulaglutide 1.5 mg once a week, general patient data, primary and secondary observation results and exploratory indicators were collected once again. In addition, blood specimens from the patients were retained for further analysis.

### 2.3 Sample Size Calculation

Sample size calculation was generated based on the results of a pretest measuring liver fat content. We selected 10 subjects before treatment and more than 3 months after treatment to determine the changes in liver fat content before and after treatment. The hepatic fat content of the patients before treatment was 13.83 ± 6.94, and after treatment, it was 8.21 ± 5.55. To design this study with *α* = 0.05 and 1‐β = 0.8, we used SPSS 26.0 software to calculate the required sample size. It was determined that 22 subjects should be included to ensure a statistically significant difference in the pre‐ and posttreatment comparisons. Taking into account a design release rate of 20%, the expected number of patients to be included was 28.

### 2.4 Definitions

According to the Chinese Expert Consensus on Type 2 Diabetes Remission (2021), important reference criteria for diabetes remission include BMI ≤ 24 kg/m^2^, weight loss ≥ 10 kg or weight loss ≥ 10%. In our study, we categorised the patients' weight loss into three groups: less than 5% loss (weight not reaching the standard), 5%–10% weight loss (weight partially reaching the standard) and weight loss > 10% (weight reaching the standard).

### 2.5 Clinical Data Collection and Testing

General information included age, height, weight and body mass index (BMI), which was calculated as weight (kg) divided by height squared (m^2^). Additionally, systolic and diastolic blood pressure, as well as the history of diabetes mellitus, were recorded. Blood samples were collected after an overnight fasting period. HbA1c was measured by HPLC (Bio‐Rad Variant II HbA1c analyser; Bio‐Rad, Hercules, CA, USA). Serum c‐peptide levels were measured using electrochemiluminescence (Cobas e 601; Roche Diagnosis, Mannheim, Germany). Blood glucose and lipids such as free fatty acids (FFAs), TGs, total cholesterol (TC), low‐density lipoprotein cholesterol (LDL‐C) and high‐density lipoprotein cholesterol (HDL‐C) were measured by Beckman Coulter AU 680 (Krefeld, Germany). ALT, AST, urea nitrogen, creatinine, uric acid and transaminases were measured using an automatic biochemical analyser (TBA‐40FR, Toshiba Company, Japan). Liver fat content was measured using a medical magnetic resonance imaging (MRI) system (3.0 T) (MAENTOM PRISMA, Siemens, Germany). The measurement of hepatic fat content was performed by the same technician in a fixed plane of the liver.

Biological samples of serum were collected by procoagulation tubes and placed in a refrigerator at 4°C for 4 h. Then, they were centrifuged at 2°C–8°C for 20 min at 1000 *g*, and the supernatant was dispensed into 0.5‐mL centrifuge tubes. These tubes were quickly placed in a refrigerator at −80°C for freezing and preservation. FGF21 and lipocalin were measured using an ELISA Kit (Elabscience E‐EL‐H0074; E‐TSEL‐H0020).

### 2.6 Statistical Methods

Statistical analyses were performed using SPSS software version 26.0 (SPSS IBM Corporation, Armonk, NY, USA), and GraphPad 8.0.2 was applied for bar graphing. Normally distributed continuous variables were expressed as mean ± standard deviation (SD), non‐normally distributed continuous variables as median (interquartile range, IQR) and categorical variables as frequency (%). For statistical analyses between treatment groups, differences between groups of normally distributed variables were analysed using paired *t*‐tests; differences between non‐normally distributed variables were analysed using paired nonparametric tests. Statistical analysis between treatment and normal control groups and differences between groups of normally distributed variables were tested using ANOVA and Kruskal–Wallis test for non‐normally distributed variables.

## 3 Results

Table 1 shows the differences in demographics as well as primary and secondary observables between the patient groups before and after GLP‐1RA treatment. The average age of the patients was 39 years, with an average disease duration of 2 years, and a predominance of male patients.

**TABLE 1:** General conditions before and after GLP‐1 treatment.

	Before treatment (*n* = 38)	Post treatment (*n* = 38)	*p*
Sex (male)	32 (84%)	32 (84%)	
Age (years)	39.55 ± 11.58	39.55 ± 11.58	
Duration of diabetes (years)	2.00 (0, 14.00)	2.00 (0, 14)	
Weight (kg)	92.29 ± 14.94	87.35 ± 14.09	< 0.05
BMI (kg/m^2^)	29.35 (25.56, 47.97)	28.56 (22.86, 44.84)	< 0.05
Systolic blood pressure (mmHg)	133 (85, 177)	130 (88, 169)	0.346
Diastolic blood pressure (mmHg)	85 (60, 122)	86 (62, 135)	0.935
FGF21 (pg/mL)	127.94 (15.46, 455.94)	169.60 (8.61, 660.23)	0.013
Lipocalin (ng/mL)	15,450 (3106, 51,415)	20,783 (3575, 58,164)	< 0.001***
Liver fat content (%)	12.11 (3.20, 30.10)	8.40 (3.4, 27.2)	< 0.001***
Fasting blood glucose (mmol/L)	7.80 (4.43, 17.17)	6.19 (4.07, 14.00)	0.004
HbA1c (%)	8.20 (5.10, 15.50)	6.60 (5, 13.50)	0.001
c‐0 (ng/mL)	3.21 (0.98, 8.30)	3.02 (1.65, 8.20)	0.973
c‐60 (ng/mL)	5.61 ± 2.28	6.87 ± 3.10	0.106
c‐120 (ng/mL)	6.73 (2.17, 16.10)	6.59 (3.43, 19.20)	0.493
Insulin Resistance Index	1.51 (1.50, 1.53)	1.51 (1.50, 1.53)	0.039
Area under the c‐peptide curve	12.74 (6.60, 27.80)	10.81 (4.20, 23.40)	0.176
ALT (U/L)	25.00 (9.00, 119.00)	19.50 (13, 167)	0.065
AST (U/L)	33.00 (10.00, 179.00)	32.00 (17, 109)	0.173
Urea nitrogen (mmol/L)	5.75 (3.04, 35.00)	5.01 (3.70, 9.59)	0.767
Cr (μmol/L)	57.00 (42.00, 202.50)	59.00 (39.00, 180.00)	0.683
eGFR (mL/min/1.73 m^2^)	115.30 (36.40, 148.30)	119.10 (42.00, 156.40)	0.818
Uric acid (μmol/L)	388 (227, 572)	361 (265, 638)	0.472
FFA (mmol/L)	0.50 (0.11, 0.80)	0.46 (0.22, 1.03)	0.343
TG (mmol/L)	2.15 (0.75, 11.15)	2.51 (0.96, 9.96)	0.6
TC (mmol/L)	5.001 ± 1.45	4.69 ± 0.96	0.244
LDL‐C (mmol/L)	3.01 ± 1.33	2.58 ± 0.84	0.046
HDL‐C (mmol/L)	1.04 (0.64, 1.34)	1.01 (0.78, 2.08)	0.224
GLP‐1	100%	80.56%	0.059
Metformin (%)	63.89%	47.22%	0.083
α‐Glucosidase inhibitor (%)	11.10%	13.88%	0.564
SGLT‐2 (%)	27.78%	19.44%	0.317
Thiazolidinedione (%)	41.67%	30.56%	0.248
DPP‐4i (%)	11.10%	8.33%	0.564
Insulin (%)	8.33%	8.33%	1

*Note:* All changes are from baseline to 3 months. Plus–minus values are mean ± SD. The eGFR calculation is based on the MDRD equation.

Abbreviation: eGFR, estimated glomerular filtration rate.

*** *p* < 0.001 by t‐test.

It is evident that after 3 months of treatment, the patients' BMI significantly decreased compared to before treatment. The main observation index of liver fat content also significantly decreased from 12.11% to 8.4% (*p* < 0.05). The rate of blood glucose compliance, as measured by glycosylated haemoglobin, increased from 69% before treatment to 48.28% after treatment (*p* < 0.05). According to the cut‐off points of 5% and 10% weight reduction, the rate of partial weight remission was 28.57%, complete remission was 17.14% and no remission was observed in 54.29% of the patients. Additionally, FGF21 levels (158.73 pg/mL vs. 169.60 pg/mL, *p* = 0.013) and lipocalin levels (15,450 ng/mL vs. 20,783 ng/mL, *p* < 0.05) were significantly elevated.

After treatment, fasting blood glucose and glycosylated haemoglobin showed improved control compared to before treatment. Considering that some patients were receiving insulin treatment and that c‐peptide and insulin are released in equal amounts, we used c‐peptide to calculate the insulin resistance index. We can see the insulin resistance index significantly decreased compared to before treatment. Among the biochemistry‐related indices, there was statistical significance observed for a significant decrease in LDL‐C and glutathione levels.

We calculated the change in liver fat content after GLP‐1RA treatment and divided the patients into two subgroups based on the magnitude of the change: Q1 group (average decrease in liver fat content of 0.37%) and Q2 group (average decrease in liver fat content of 8.6%). It is evident that the Q2 group, which had a higher degree of decrease in liver fat content, had a higher initial liver fat content compared to the Q1 group (15.95% vs. 9.47%, *p* < 0.05). However, there were no significant differences between the two groups in terms of patients' age, disease duration, body weight, glycaemic control and pancreatic islet function. Based on the Chinese Expert Consensus on the Remission of Type 2 Diabetes (2021), which categorises patients into weight not in remission, partial remission and complete remission using weight loss of 5% and 10% as cutoff points, the Q1 group had 38.89% partial weight remission and 0% complete weight remission, while the Q2 group had 44.44% partial weight remission and 22.22% complete weight remission respectively.

After treatment, compared to the Q1 group, the Q2 group exhibited lower fasting blood glucose levels and glycated haemoglobin levels (*p* = 0.018; *p* = 0.094), as well as a better glycaemic control rate (*p* = 0.041). Following 3 months of GLP‐1RA treatment, the Q2 group demonstrated an average decrease in liver fat content of 8.6%, a percentage decrease in glycated haemoglobin of 18.17%, a percentage decrease in body weight of 7.29% and an increase in c‐peptide release of 1.03%, 28.86% and 18.28% at fasting, 1 and 2 h respectively. In contrast, the Q1 group experienced an average decrease in liver fat content of only 0.37%, a percentage decrease in glycated haemoglobin of 6.53%, a decrease in body weight of 3.41%, a decrease in fasting c‐peptide release of 1.91% and an increase in 1‐h and 2‐h c‐peptide release of 19.18% and 11.66% respectively.

## 4 Discussion

In cases of diabetic obesity, the release of lipids, primarily TGs, from adipose tissue is associated with an enlargement of skeletal muscle, liver and other organs and tissues [13]. As weight increases, excess lipids escape into various visceral structures, and the accumulation of visceral adipose tissue can lead to numerous serious consequences. Omental and mesenteric fat, in particular, are associated with metabolic disorders and adverse outcomes related to obesity [14]. Perirenal adipose tissue, on the other hand, exerts pressure on the kidneys, potentially activating the RAAS system, causing renal injury and complicating the treatment of hypertension [15]. Elevated levels of FFAs and inflammatory cytokines in nonadipose tissues can disrupt insulin signalling, leading to insulin resistance [16]. Insulin resistance is strongly linked to excessive abdominal adipose tissue [2].

Type 2 diabetes, particularly when combined with obesity and fatty liver disease, can be reversible to some extent, contrary to the common perception of it being a lifelong condition. However, the natural remission and reversal rates of Type 2 diabetes are generally very low [17]. The rate of partial reversal is 1.5%, complete reversal is 0.14% and long‐term reversal is only 0.01%. Therefore, an intensive intervention programme is necessary to achieve reversal.

It is now widely recognised that weight loss plays a central role in reversing T2DM, and the double‐circulation doctrine provides the rationale that weight loss can alleviate or even reverse diabetes to some extent [18]. Moderate weight loss, defined as a 5%–10% reduction in baseline weight, is associated with clinically significant improvement in obesity‐related metabolic risk factors and coexisting conditions. Weight loss reduces insulin resistance, improves pancreatic beta‐cell function and enhances hepatic and skeletal muscle sensitivity to insulin. Currently, the main methods used for weight loss include lifestyle interventions such as exercise and diet, surgical treatments and weight‐loss glucose‐lowering medication‐assisted therapy. In the Qatari DIADM‐I Trial [19], which targeted 147 obese patients with T2DM, the diet‐controlled, exercise‐supported group showed a mean weight loss of 11.98 kg (95% CI 9.72–14.23) after 12 months of treatment, compared to 3.98 kg (2.78–5.18) in the control group. In the intervention group, 21% of participants achieved a weight loss of more than 15% of their body weight between baseline and 12 months, while only 1% in the control group achieved the same (*p* < 0.0001). However, for patients with a BMI ≥ 35, lifestyle interventions alone, such as diet and exercise, were not found to be beneficial, and surgical intervention (gastric bypass or sleeve gastrectomy) was recommended.

GLP‐1RA, recommended as a first‐line drug by the ADA guidelines for the treatment of obese patients with T2DM and comorbidities, is now widely utilised in clinical practice [20]. It has also received approval in the United States for the treatment of NAFLD [21]. GLP‐1RA has been extensively studied both in basic research and clinical trials. These studies have demonstrated that aside from its insulinotropic effects at physiological concentrations, GLP‐1 at pharmacological concentrations can also reduce appetite, decrease food intake and effectively manage long‐term body weight. However, while the combination of MRI techniques to quantify the effects of GLP‐1 has shown promise in reducing appetite, curbing unintentional intake and controlling long‐term body weight [22], limited studies have explored the relationship between changes in liver fat content and the degree of remission of diabetes mellitus and comorbidities. Moreover, there is a lack of research investigating the quantitative determination of liver fat content using MRI techniques in conjunction with these aspects.

The gold standard for assessing fatty liver is liver biopsy, which involves taking multiple tissue samples to accurately measure liver fat content. However, liver biopsies are difficult to perform clinically and require multiple follow‐ups, making them unsuitable as routine tests. As a result, MRI is used as an alternative method, which has been found to be effective in evaluating NAFLD. In MRI, the number of hydrogen atoms in lipids and water molecules is calculated using the magnetic properties of hydrogen protons in a specific magnetic field. The proton density fat fraction (PDFF) is then calculated as the ratio of fat proton density to the total proton density (including both fat and water) [23]. Quantifying liver fat using PDFF is more advantageous than liver biopsy, especially when monitoring changes in liver fat content over time [12]. Therefore, PDFF is a reliable method for quantifying liver fat.

Our study found that GLP‐1 significantly reduced hepatic fat content, resulting in a mean decrease of 3.71% after 3 months of treatment. In the Indian D‐LIFT study [24], dulaglutide application for 24 weeks led to an absolute change in hepatic fat content of 3.5% (95% CI −6.6, −0.4; *p* = 0.025) and a relative change of 26.4% compared to placebo (−44.2, −8.6; *p* = 0.004), representing a 2.6‐fold decrease. Another study conducted in China with 96 subjects [25] randomised patients to receive treatment with glucagon, liraglutide or placebo (1:1:1). After 26 weeks of treatment, both the liraglutide and glucagon groups showed a significant reduction in intrahepatic fat content compared to the placebo group (liraglutide 26.4% ± 3.2% to 20.6% ± 3.9%, *p* < 0.05; glucagon 25.0% ± 4.3% to 22.6% ± 5.8%, *p* > 0.05). The difference in results between our study and others may be attributed to the relatively short duration of our treatment period on one hand and the possibility of reaching a plateau phase by the patients on the other hand. As shown in Table 2 for the Q1 and Q2 groups, the initial mean hepatic fat content levels were 9.47% and 15.95%, respectively, and there was a statistically significant difference between the groups (*p* < 0.05). After 3 months of treatment, the mean hepatic fat content in the Q1 and Q2 groups was 8.4% and 8.09%, respectively, and the difference between the groups disappeared (*p* = 0.767). This suggests that there may be a therapeutic plateau reached by the presence of GLP‐1RA in reducing hepatic fat content to around 8%. However, further trials are needed to validate this observation. Furthermore, our study demonstrated that patients with higher initial hepatic fat content achieved a greater reduction in hepatic fat content when treated with GLP‐1RA (0.37% vs. 8.6%, *p* < 0.05). Our study found that patients with higher hepatic fat content experienced greater reductions in fat‐containing content and showed better improvements in glycated haemoglobin levels and recovery of pancreatic islet function after 3 months of treatment. Although there were no significant changes in fasting c‐peptide secretion levels in the Q1 and Q2 groups, the Q2 group exhibited a 28.86% increase in 1‐h c‐peptide release and an 18.28% increase in 2‐h c‐peptide release. In comparison, the Q1 group showed a 19.18% increase in 1‐h c‐peptide release and an 11.66% increase in 2‐h c‐peptide release. These findings suggest that reducing hepatic fat content through GLP‐1RA treatment may facilitate the recovery of islet function in the presence of pancreatic islets.

**TABLE 2:** Grouping according to the degree of decrease in liver fat content.

	Group Q1 (*n* = 18)	Group Q2 (*n* = 18)	*p*
Age (years)	40.00 ± 13.58	39.06 ± 10.06	0.814
Disease duration (years)	3.08 ± 4.00	2.54 ± 2.86	0.838
Decrease in liver fat content (%)	0.37 ± 2.80	8.6 ± 5.00	< 0.001
Pretreatment			
Weight (kg)	88.50 (80.00, 148.60)	90.50 (68, 120)	0.815
Liver fat content (%)	9.47 (3.20, 27.50)	15.95 (8.50, 30.10)	0.001
FGF21 (pg/mL)	67.53 (15.46, 455.90)	140.00 (21.90, 299.50)	0.52
Lipocalin (ng/mL)	15,304 (5424, 51,418)	15,806 (3106, 43,420)	0.767
Fasting blood glucose (mmol/L)	9.40 ± 6.93	7.49 ± 2.58	0.085
HbA1c (%)	8.98 ± 2.14	8.19 ± 2.04	0.287
c‐0 (ng/mL)	3.23 (1.94, 7.88)	3.11 (1.63, 8.3)	0.304
c‐60 (ng/mL)	5.79 ± 2.18	5.65 ± 2.45##	0.865
c‐120 (ng/mL)	6.92 ± 2.92	7.59 ± 3.61##	0.553
Posttreatment			
Weight (kg)	87.00 (75.00, 142.00)	82.50 (60.00, 101.00)	0.323
Liver fat content (%)	8.40 (3.70, 27.2)	8.09 (3.40, 18.6)	0.767
FGF21 (pg/mL)	188.10 (47.72, 435.10)	172.60 (20.47, 642.10)	0.446
Lipocalin (ng/mL)	29,938 ± 18,226	22,829 ± 10,399	0.162
Fasting blood glucose (mmol/L)	8.05 ± 2.88	5.94 ± 1.36	0.018
HbA1c (%)	7.72 ± 2.49	6.41 ± 1.00	0.094
c‐0 (ng/mL)	4.11 ± 1.86	3.76 ± 1.76	0.573
c‐60 (ng/mL)	6.51 ± 2.97	7.17 ± 3.33##	0.583
c‐120 (ng/mL)	7.46 ± 3.79	8.67 ± 4.31	0.444
Percentage of weight loss	3.41%	7.29%	
Increase in FGF21 (%)	164.29%	64.21%	
Lipocalin increase (%)	60.81%	64.67%	

*Note:* All changes are from baseline to 3 months.

^##^ Q2 group had significantly higher c‐peptide 1 h postprandial after treatment, *p* = 0.018.

Furthermore, we observed an interesting phenomenon during the treatment period. As in the case of weight loss with a plateau period, some patients, even though their initial hepatic fat content varied considerably, concentrated their hepatic fat content at about 8% after treatment with GLP‐1RA. The reason for the plateau period is unclear, and we have made some speculations. GLP‐1RA reduces hepatic fat content by decreasing the expression of the carbohydrate‐responsive element‐binding protein (ChREBP) transcription factor, which inhibits fat synthesis [26]. While silencing ChREBP promotes lower hepatic TG levels and less hepatic steatosis, it can in turn lead to enhanced cholesterol synthesis [27]. On the flip side, the current belief is that weight rebound after weight loss may be due to adipocyte atrophy, which inhibits lipolysis and the release of fatty acids from adipocytes due to the lack of remodelling of the extracellular matrix, which creates mechanical stress between the cells and the oversized extracellular matrix [28]. Although there is no direct evidence of changes in steatotic hepatocytes during atrophy, we speculate that the rapid removal of hepatic fat resulting from the application of GLP‐1RA may result in hepatocytes exhibiting the same pathology.

GLP‐1RA has been found to significantly reduce liver enzyme markers and LDL‐C levels, while also decreasing liver fat content. Obese patients often experience comorbidities related to abnormal liver function, which can be attributed to dysfunction in visceral fat, leading to local steatosis, hypoxia, inflammation, endoplasmic reticulum stress and fibrosis [29, 30]. Studies have shown that GLP‐1RA analogues can mitigate obesity‐induced liver damage and restore liver function [31]. However, the precise mechanism behind this effect has not been fully elucidated and may involve multiple pathways aimed at attenuating oxidative stress induced by lipotoxicity [32, 33]. Additionally, FGF21 and lipocalin have been identified as regulators of lipid metabolism that can help reduce hepatic lipotoxicity.

FGF21 is an endocrine hormone that independently regulates glucose and lipid metabolism. In our study, we observed that patients had significantly higher FGF21 levels compared to normal control patients prior to GLP‐1RA treatment. Interestingly, after GLP‐1RA treatment and subsequent weight loss in patients, FGF21 levels did not decrease but rather increased significantly (*p* < 0.05). This finding appears paradoxical since FGF21 levels are typically low in nonobese individuals but significantly elevated in cases of obesity, insulin resistance and diabetes mellitus [34]. We propose two hypotheses to explain these observations. Firstly, it is possible that the patients in the treatment group did not achieve complete weight loss and glycaemic control following GLP‐1RA treatment, leading to compensatory increase in FGF21 levels. Secondly, it is plausible that GLP‐1 directly stimulates the secretion of FGF21. Previous studies have indicated that FGF21 secretion is notably higher in obese diabetic patients after exenatide treatment [35]. In diabetic mice studies, it has been shown that GLP‐1RA stimulates FGF21 secretion in hepatocytes. Conversely, Glp1r^−/−^ mice lacking the GLP‐1 receptor did not exhibit weight reduction or regulation of lipid metabolism and failed to stimulate FGF21 expression [36]. Inhibition of FGF21 expression attenuated the inhibitory effect of GLP‐1 on hepatic glucose export [37]. Similarly, the weight‐reducing and lipid metabolism‐regulating effects of GLP‐1RA were significantly diminished or absent in hepatic FGF21 knockout mice [36]. These findings suggest that GLP‐1 can regulate glucose metabolism by upregulating FGF21 expression, and FGF21 is necessary for GLP‐1RA to exert certain physiological effects. However, it should be noted that in healthy individuals with lean body mass, changes in GLP‐1 levels do not seem to cause corresponding changes in FGF21 levels [38].

Lipocalin is an endogenous hormone secreted by adipocytes and acts as an insulin sensitiser. In our study, we observed that all patients in the treatment group had significantly lower levels of lipocalin compared to normal control patients. Even after GLP‐1RA treatment, lipocalin levels increased significantly but did not return to normal levels. Lipocalin, being secreted by adipose tissue, has various sites of action in different organs. Elevated lipocalin levels effectively prevent liver and kidney fibrosis [39], promote renal function recovery and facilitate β‐cell reconstruction [40]. The physiological effects of lipocalin may be mediated by reducing ceramide concentrations and activating the downstream phospholipase C–inositol triphosphate–calcium ion pathway [41]. High ceramide concentrations inhibit insulin signalling, while reduction of hepatic ceramide concentrations restores insulin resistance [42, 43]. This mechanism increases glucose uptake by muscle and adipose tissue. GLP‐1RA can regulate lipocalin expression via Sirt1 and the transcription factor Foxo‐1 [44]. Blocking Sirt1 and Foxo‐1 significantly inhibits GLP‐1RA‐induced lipocalin expression. Therefore, GLP‐1RA may improve insulin resistance and promote recovery of pancreatic islet function through the regulation of lipocalin expression.

In conclusion, GLP‐1RA can achieve diabetic blood glucose control by reducing liver fat content, decreasing insulin resistance and restoring functional pancreatic secretion. This study is a multicentre, open‐label, self‐controlled study. However, we also included a normal control group consisting of nondiabetic individuals without fatty liver, allowing us to compare and assess the treatment effects more effectively. Additionally, we added exploratory measures such as FGF21 and lipocalin based on previous clinical studies to provide a comprehensive evaluation. We ensured strict adherence to information and specimen collection requirements to enhance the authenticity and reliability of our results. Patients were recruited from both outpatient and inpatient settings at two centres, which helped minimise selection bias to some extent.

However, there are certain limitations to our study. Firstly, the study population was limited to a specific ethnic group, so the results may not be representative of diabetic patients from other populations. The 38 patients did not include elderly patients, so these data do not reflect the situation of elderly diabetic patients. Secondly, although there was a decrease in the mean and median number of medications after GLP‐1RA treatment, it did not reach statistical significance. This may be attributed to the relatively short treatment duration in our study. Therefore, further clinical studies with longer follow‐up periods are needed to provide additional evidence and strengthen our findings.

## Author Contributions

We thank all participants in this study. C.L. performed the statistical analysis and wrote the first draft of the manuscript. Y.H. and Y.X. contributed to the discussion and collected the data. L.X. and R.Z. revised the manuscript. R.Z. perform imaging examination procedures. Y.W. and W.W. are corresponding author.

## Ethics Statement

Approval of the research protocol: We obtained ethical approval from Qingdao University Hospital. Approval date of Registry and the Registration No. of the study/trial: QYFY WZLL 25617.

## Consent

Informed consent: We obtained informed consent from enrolled patients.

## Conflicts of Interest

The authors declare no conflicts of interest.

## Data Availability

The datasets generated and/or analysed during the current study are not publicly available due to our next research but are available from the corresponding author upon reasonable request.
